# From ball-milling to bead-milling technology: rapid optimization and scale-up of a one-pot Wittig olefination–Diels–Alder reaction sequence using an agitator bead mill

**DOI:** 10.1039/d6mr00016a

**Published:** 2026-07-07

**Authors:** Nina Biedermann, Johanna Templ, Kenneth Banderob, Philippe M. C. Roth, Michael Schnürch

**Affiliations:** a Institute of Applied Synthetic Chemistry, TU Wien Getreidemarkt 9/163 1060 Vienna Austria michel.schnuerch@tuwien.ac.at; b Willy A. Bachofen AG Junkermattstrasse 11 4132 Muttenz Switzerland

## Abstract

Mechanochemical methods offer a sustainable alternative to traditional solution-based synthesis, yet their scalability beyond laboratory-scale ball mills remains a challenge and often requires lengthy re-optimization for larger scales. Herein, we report the rapid batch translation of a one-pot Wittig olefination–Diels–Alder reaction sequence from a ball mill to an agitator bead mill (DYNO®-MILL RESEARCH LAB), achieving a scale-up from milligram to multi-gram quantities. Crucially, this translation was accomplished with minimal on-the-fly optimization, requiring only a few bead-milling runs to identify suitable operating conditions and perform the transformation in batch mode with good efficiency. Our results provide a practical case study for the scalability of mechanochemical reactions by using bead-milling technology and highlight the potential of bead-milling devices for future process development.

## Abstract 2

A rapid bead milling scale-up protocol of a one-pot Wittig olefination–Diels–Alder cycloaddition sequence with complete endo-selectivity is reported.

## Graphical abstract

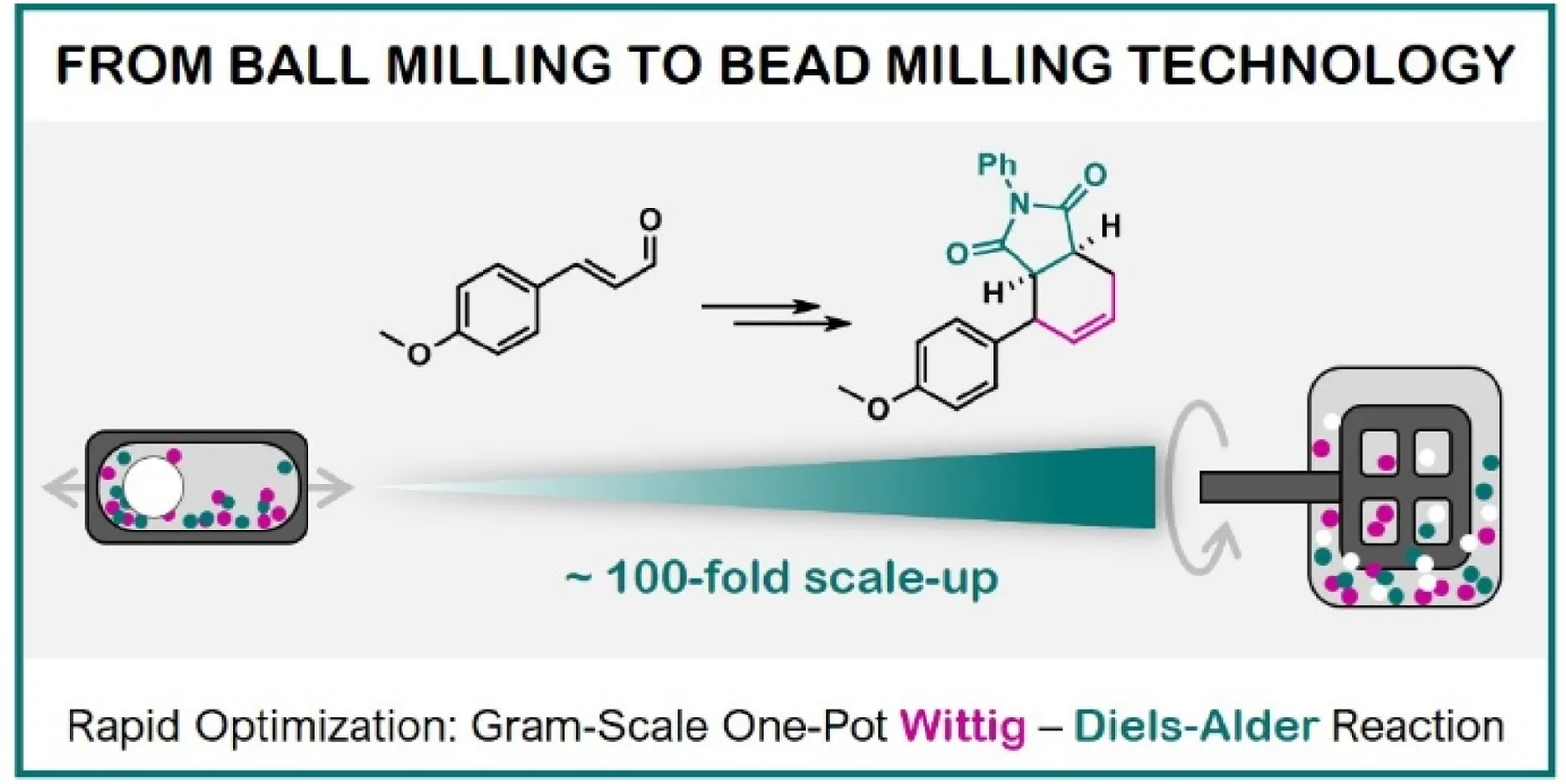

## Introduction

Mechanochemical synthesis has emerged as a powerful alternative to solution-based chemistry, offering numerous advantages, including reduced solvent consumption and waste generation, simplified operation, and access to unique reactivity.^1–4^ Despite these benefits, the scalability of mechanochemical protocols developed using laboratory-scale ball milling equipment remains a significant challenge.^5,6^ In this context, agitator bead mills represent promising tools, particularly for continuous processing. In agitator bead mills, energy input is achieved by high-speed rotation of a grinding chamber containing a large number of microbeads, enabling efficient energy transfer through shear, friction, and impact. Historically, bead milling has been widely applied in wet processing and formulation, particularly for particle size reduction and dispersion, including the preparation of nanoparticles,^7^ pigment dispersion,^8^ and the processing and formulation of pharmaceuticals^9–11^ and nutritional additives.^12,13^ However, despite their industrial use, agitator bead mills have rarely been investigated and used for chemical synthesis at scale, particularly for mechanochemical organic transformations.^14–23^

Recent studies have nevertheless demonstrated the potential of bead-milling devices such as the high-throughput reaction platforms developed by Willy A. Bachofen AG (WAB-GROUP®) for organic synthesis at scale. Reported examples include the mechanochemical batch synthesis of acetaminophen (paracetamol) *via* Beckmann rearrangement,^14^ a scalable continuous-flow mechanochemical synthesis of amides *via* EDC coupling,^15^ the scale-up of nitration reactions of alcohols and arenes,^16^*N*-arylation of sulfonamides *via* C–N coupling,^17^ and the continuous-flow production of vanillin.^18^ In addition, semi-continuous mechanochemical processes have been demonstrated for the production of biodiesel^19^ and the isomerization of glucose to fructose.^20^ Further studies have explored continuous-flow heated mechanochemistry for the acetylation of glycol,^21^ the synthesis of deep eutectic solvents,^22^ and solketal synthesis.^23^ It should be noted that while other horizontal bead-milling devices are available (*e.g.*, Netzsch, Bühler), these systems are typically designed for substantially larger industrial scales and conventional wet-processing applications, and have not been reported for mechanochemical synthesis.

**Fig. 1:**
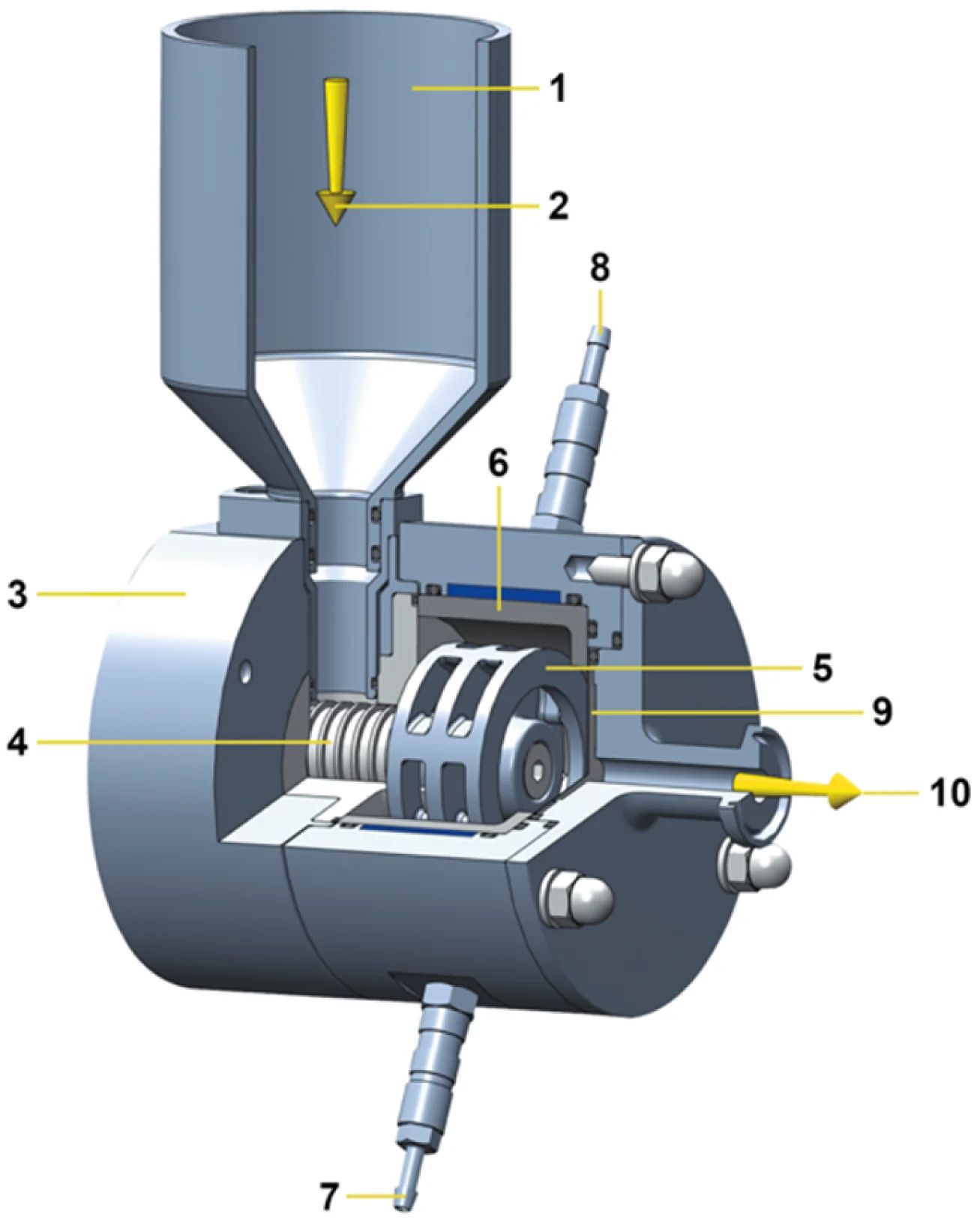
Schematic illustration of the DYNO®-MILL (agitator bead mill). Legend: (1) – feed funnel; (2) – product inlet; (3) – seal housing for lip seal or double mechanical seal; (4) – feed screw; (5) – DYNO®-ACCELERATOR; (6) – coolable grinding container; (7) – cooling water inlet; (8) – cooling water outlet; (9) – grinding beads separator (sieve or screen plate); (10) – product outlet. Altered image reproduced with permission from Willy A. Bachofen AG, Switzerland.

The DYNO®-MILL, an agitator bead mill, offers a particularly attractive platform for performing mechanochemical transformations at scale, either in batch, semi-continuous, or continuous mode. The primary advantage of this platform lies in its deep industrial maturity; rather than requiring the development of novel scaling architectures, DYNO®-MILLs represent a well-established infrastructure utilized for large-scale wet processing. Repurposing this existing commercial machinery for organic synthesis ultimately offers an attractive platform for multi-kilogram scale-up of mechanochemical transformation. It features a cylindrical grinding chamber and the patented DYNO®-ACCELERATOR, which is mounted on a rotating shaft connected to a motor (see Fig. 1). The chamber can be fed either through a feed funnel and screw (semi-continuous or continuous mode) or through the outlet lid by unscrewing the sieve (in continuous mode) or screen plate (in batch mode). In addition, the device features a cooling water circuit for the grinding chamber, enabling active temperature regulation of the reaction mixture. This offers the possibility to exclude thermal effects, ensuring that reactions are solely driven by mechanical activation and allowing the use of heat-sensitive compounds, which might decompose or give side reactions in the absence of cooling. Through high-speed rotation of the accelerator, the chemicals and grinding media collide, providing high-energy input *via* shear, friction, impact, and shock. In bead milling, solid reactants are subjected to mechanical activation through compressive stress arising from compression, as well as impact stress by single-bead collisions. The use of a large number of grinding beads increases both the frequency of stress events and the amount of energy transferred per event. Therefore, the efficiency of the mechanochemical process is determined by the probability that reactant particles are trapped in energetically active grinding zones and interact with beads carrying sufficient mechanical energy to induce reactivity.^24^ Hence, the amount of beads, typically reported in grams and total volume fraction of the reaction chamber, is a critical parameter to be optimized when carrying out a given transformation in a bead mill. Furthermore, the tip speed (given in m s^−1^ or rpm) defines the kinetic energy of the beads and is another critical parameter. These two parameters can be considered as the “bead mill equivalents” of the number and weight of milling balls as well as frequency in ball mills.

Herein, we report the rapid translation of a one-pot Wittig olefination–Diels–Alder sequence from a ball mill to a DYNO®-MILL employing bead-milling technology, achieving a scale-up from milligram to multi-gram quantities. Although the DYNO®-MILL offers operation in continuous mode, all experiments in this study were conducted in batch mode. Notably, the transformation proceeded with good efficiency without extensive optimization of the reaction parameters, demonstrating the potential of agitator bead mills to allow the rapid and straightforward scale-up of mechanochemical reactions.

## Results and discussion

The aim of this study was to scale up ball-milling experiments in a short period of time without extensive optimization. Increasing the scale from milligram to gram quantities is often an important step in the development of active pharmaceutical ingredients to allow more studies (*e.g.*, toxicology, pharmacokinetics) before a compound qualifies for the next milestone, *i.e.*, clinical studies. Hence, at this stage, lengthy optimization of reaction parameters is not undertaken; it is more important to obtain the desired material in sufficient quantity quickly. Under this prerequisite, the following results were obtained.

### One-pot Wittig olefination–Diels–Alder sequence

All experiments described herein were conducted using the DYNO®-MILL RESEARCH LAB operated in batch mode, featuring a grinding chamber with an inner cylinder (*V* = 80 mL) and the DYNO®-ACCELERATOR both made from hardened stainless steel (for pictures, see SI). The batch-mode setup was deliberately selected to mirror the operational regime of the original ball-milling protocol. In this configuration, the feed funnel was removed, a screen plate was installed at the outlet lid, and all solid reactants and reagents were charged directly into the grinding chamber by unscrewing the outlet lid. Operationally, the sequential one-pot procedure was executed by turning off the mill at the end of each step, unscrewing the reaction chamber from the motor unit, and placing the chamber upright to unscrew the outlet lid. The solid reagents (NH_4_Cl or dienophile 3) were directly added to the static bed of beads and reaction mixture. The lid was then re-secured, the chamber reattached to the motor unit in its horizontal operating position, and milling resumed. No intermediate material transfer or bead separation was required between the individual reaction steps.

**Scheme 1:**
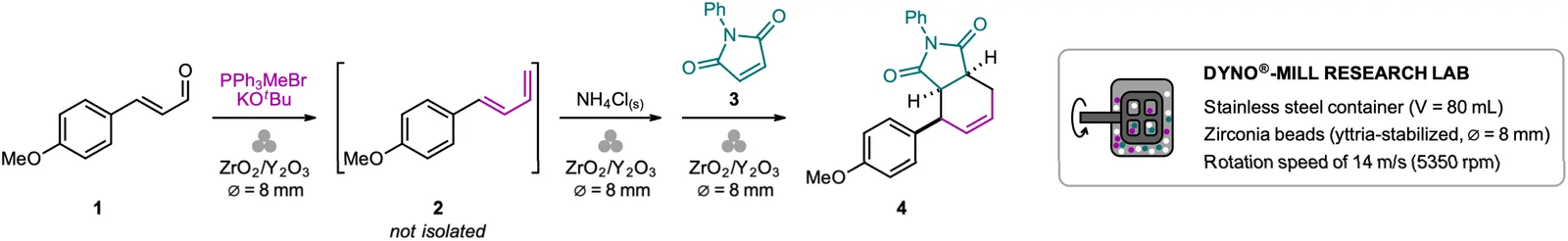
One-pot Wittig olefination–Diels–Alder reaction sequence, which was investigated in a DYNO®-MILL RESEARCH LAB using ZrO_2_/Y_2_O_3_ beads at scale.

Our experiments focused on the direct transfer of the reported one-pot Wittig–Diels–Alder ball-milling protocol to the agitator bead mill at increased scale (Scheme 1). A first attempt at a higher substrate loading (10 g of 1) resulted in incomplete conversion and handling issues, which were attributed to overfilling of the grinding chamber and resulting in insufficient mixing efficiency. In a next attempt, the substrate amount was reduced to 5.0 g, which led to a marked improvement in reaction performance (Table 1). Aldehyde 1 (5.0 g), Wittig salt and base, using the same stoichiometric amounts as previously reported,^25^ were filled into the grinding chamber (*V* = 80 mL), which contained yttria-stabilized zirconia beads (91 g, *φ* = 0.30). Initially, milling at a tip speed of 10 m s^−1^ (3820 rpm) was performed for 5 minutes; however, only minor conversion to the olefination intermediate 2 was observed. The accelerator speed was increased to 14 m s^−1^ (5350 rpm) and grinding continued for 5 minutes, still not resulting in sufficient conversion. Given the reduced batch size compared to the initial experiment, insufficient mixing and mechanical impact were expected. Therefore, further beads were added to the grinding chamber, resulting in a total bead mass of 159 g (*φ* = 0.525). While this adjustment effectively filled the practical volume of the grinding chamber, we remained well within the typical bead-filling volumes for agitator bead mills, ranging from 30% to 80%. However, this parameter requires user experience and optimization. The underlying rationale for increasing the number of beads is that more beads increase the energy input. Crucially, even with a high filling volume, a substantial void volume remains between the individual beads. In batch mode, maintaining this void space is essential to ensure efficient mixing and bead motion, a fact considered here.

**Table 1:** Process steps and parameter adjustments for the on-the-fly optimization run at ambient conditions. Optimizations were carried out sequentially in this run during the translation of the Wittig olefination–Diels–Alder reaction sequence to bead milling under ambient conditions. The complete table, including additional process details, is provided in the SI (Table S1)

Step no.	Reaction stage	Total process time [min]	Step duration [min]	Bead mass & total volume fraction [g, *φ*]	Tip speed [m s^−1^]	Comment
1	Wittig olefination	5	5	91, 0.30	10	Initial charging and grinding of Wittig olefination reagents: aldehyde 1, PPh_3_MeBr, KO^*t*^Bu.
2	Wittig olefination	10	5	91, 0.30	14	Reaction incomplete; tip speed was raised to 14 m s^−1^ and grinding was prolonged by 5 min.
3	Wittig olefination	15	5	91, 0.30	14	Reaction incomplete; addition of beads (+68 g) (cumulative totals: 159 g, *φ* = 0.525); grinding was prolonged by 5 min.
4	Wittig olefination	20	5	159, 0.525	14	Reaction incomplete; grinding prolonged by 5 min without changes to the tip speed or bead mass.
5	Base quench	25	5	159, 0.525	14	Base quenching *via* addition of NH_4_Cl followed by 5 min of grinding.
6	Diels–Alder reaction	55	30	159, 0.525	14	Diels–Alder reaction: addition of *N*-phenylmaleimide (3) followed by grinding for 30 min.
7	Diels–Alder reaction	70	15	159, 0.525	14	Reaction incomplete; grinding was prolonged by 15 min until complete conversion of intermediate 2 was observed.

After prolonging the milling time by an additional 10 minutes, all starting material was consumed, resulting in a brown-to-orange paste (see SI). Subsequently, the base quenching step with solid ammonium chloride (NH_4_Cl) was carried out, followed by the addition of *N*-phenyl maleimide (3, 1 equiv.). After a total grinding time of 45 minutes, complete conversion in the Diels–Alder step was observed and product 4 was isolated in 48% yield upon aq. work-up and column chromatography (see Table 2, entry 3).

**Table 2:** Comparison of the scale-up experiments under bead-milling conditions and the previously reported ball-milling approach^25^ of the one-pot Wittig–Diels–Alder sequence of aldehyde 1^,^^a^

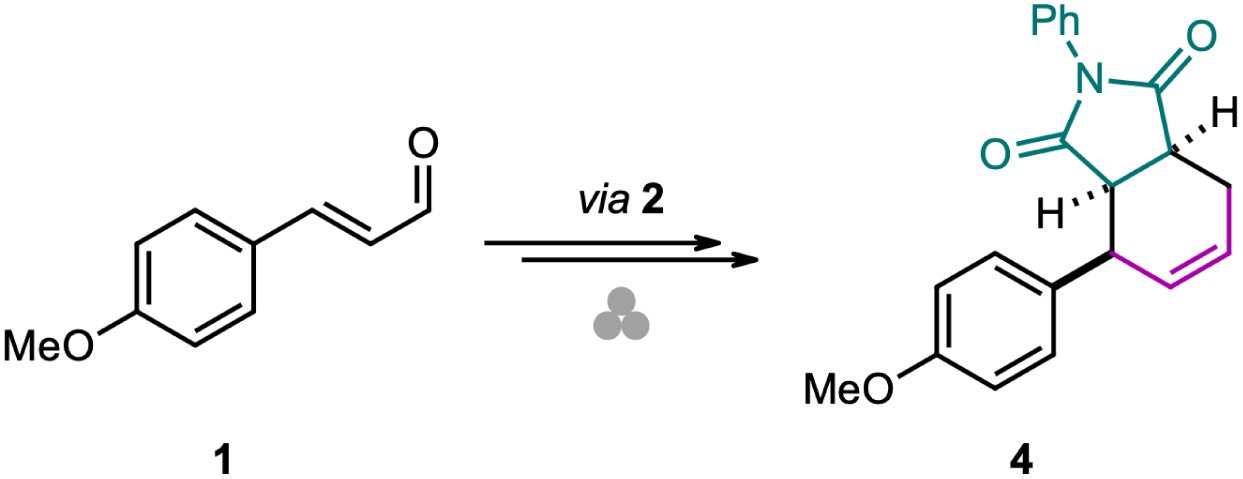							
Entry	Device	Frequency/tip speed [Hz/rpm]	Scale abs. (rel.) [mmol]	Time total [min]	Temp in/out^b^ [°C/°C]	The oretical yield of 4 [g]	Isolated yield of 4 [g/%]
1	Ball mill	36/—	0.3 (×1)	17 (32)	35.1/52.9	0.10	0.08/84
2	Ball mill	36/—	1.0 (×3)	32	—/—	0.33	0.24/73
3^c^	DYNO®-MILL	—/5350	30.2 (×100)	70	22.4/22.5	10.07	4.73/48
4^d^	DYNO®-MILL	—/5350	30.2 (×100)	45	31.6/35.4	10.07	6.72/67
5^e^	DYNO®-MILL	—/5350	30.2 (×100)	55^f^	12.3/13.8	10.07	5.99/60

a All reactions were conducted using aldehyde 1 (1.0 equiv.), PPh_3_MeBr (1.4 equiv.), KO^*t*^Bu (1.2 equiv.), NH_4_Cl (1.0 equiv.), and dienophile 3 (1.0 equiv.).

b Temperature of the reaction mixture at the beginning and end of the Diels–Alder reaction step; for ball milling experiments, temperature measurements were performed immediately after the milling process with an infrared thermometer, focusing the sensor on the stainless-steel ball in the milling vessel.^25^ For bead milling experiments (DYNO®-MILL), process temperatures were monitored with a temperature probe inserted horizontally through the outlet lid to measure the temperature of the reaction mixture/bead bed.

c Experiment performed under ambient conditions with on-the-fly optimization of process parameters.

d Experiment performed under ambient conditions with already slightly optimized reaction conditions.

e Experiment performed as entry 4 with active cooling of the reactive chamber in the Diels–Alder step.

f Reaction times of the individual steps were not changed compared to entry 4 (in total: 45 min), but an additional process step of 10 min was included after the base quenching step to allow the reaction mixture to cool down to the desired operating temperature (for details, see SI, Table S3).

Building on these findings, a refined experimental setup was investigated (details on the process steps and parameters are provided in the SI, Table S2). Right from the start, the grinding cylinder was loaded with the optimized amount of grinding beads (159 g, *φ* = 0.525), followed by aldehyde 1, Wittig salt, and base. Notably, the reaction chamber was packed sequentially in alternating layers of beads, reactants, and beads. This layered charging method was chosen because the chamber was filled to its practical capacity, ensuring optimal mixing efficiency and preventing clumping right from the outset. Milling at a tip speed of 14 m s^−1^ (5350 rpm) now led to complete conversion to intermediate 2 within 10 minutes. Consequently, solid NH_4_Cl was added and milling continued for 5 minutes. Subsequently, dienophile 3 was added, and the Diels–Alder step was performed for 30 minutes at 5350 rpm. Under these conditions, complete conversion to the desired product was achieved, and product 4 was isolated in an improved yield of 67% (6.7 g). Compared with our previously reported ball-milling approach, the reaction proceeded with comparable efficiency (67% *vs.* 84% on a 0.3 mmol scale and 73% on a 1 mmol scale). Crucially, the reaction was successfully translated to the DYNO®-MILL, requiring only minimal optimization to establish suitable operating parameters and achieve a multi-gram scale-up of 4 to 6.7 g (see Table 2, entry 4).

To further investigate the influence of temperature on the Diels–Alder step, a comparative experiment incorporating active cooling was performed. In contrast to the experiment under ambient conditions, the active cooling system was engaged during the base quenching step, which was also prolonged by 10 minutes at a reduced speed of 4 m s^−1^ to effectively cool the reaction mixture. Once the reaction mixture reached 10 °C, dienophile 3 was added, and the Diels–Alder reaction was performed for 30 minutes, maintaining the temperature below 14 °C. Under these conditions, complete conversion was again achieved, delivering product 4 in 60% (6.0 g) isolated yield (see Table 2, entry 5).

A comparison of the bead-milling experiments and the previously reported ball-milling protocol is provided in Table 2. While differences in absolute yield and reaction time are observed, the comparison highlights that the fundamental reactivity is preserved upon transfer to the agitator bead mill. These observations indicate that, despite differences in reactor design, energy input mode, and therefore generated friction heat, bead milling allowed the rapid scale-up of the investigated one-pot sequence without intensive re-optimization.

The reduced isolated yield of only 48% observed in the first bead-milling experiment (Table 2, entry 3) compared to the 67% observed in the second run (Table 2, entry 4) is attributed to the extended reaction time required during the stepwise, on-the-fly optimization of milling parameters. In this initial run, the Wittig olefination intermediate 2 was exposed to prolonged high-energy milling prior to the subsequent quenching and Diels–Alder steps. In previous studies, degradation of dienes upon extended ball milling has been observed,^25^ and is therefore likely to occur under bead-milling conditions. In contrast, the refined protocol with an adjusted bead-filling volume and rotation speed enabled rapid and complete olefination (10 minutes), followed by immediate quenching and direct transition to the cycloaddition step. Thus, the duration of mechanical impact on the intermediate and its potential degradation was minimized, resulting in a higher isolated yield.

Regarding process temperatures, significant differences were observed between the runs performed under ambient conditions without active cooling of the reaction mixture (Table 2, entries 3 and 4). In the experiment, where reaction parameters were optimized on the fly (Table 2, entry 3), the temperature during the cycloaddition step remained low (22.4–24.0 °C). Conversely, for the optimized ambient procedure (Table 2, entry 4), the starting temperature of the Diels–Alder step was significantly higher (31.6 °C), and the step concluded at an elevated temperature of 35.4 °C.

A detailed analysis of the process step logs (see SI, Table S2) reveals that this elevated thermal profile in entry 4 is initiated during the prior Wittig olefination step. Operating immediately at an optimized tip speed of 14 m s^−1^ and a bead-filling volume of 52.5% resulted in rapid heating, driving the system from 23.3 °C to 32.1 °C within the first 10 minutes. Because entry 4 was executed in a streamlined fashion without the intermittent operational stops for bead volume adjustments as in entry 3, the accumulated thermal energy was preserved through the subsequent steps. Interestingly, this significant rise in temperature during the initial Wittig olefination step was not observed for the experiment described in entry 5 (23.4 °C to 23.2 °C; for further details see SI, Table S3). The exact origin of this thermal discrepancy is not fully understood. It is tentatively hypothesized to stem from minor, unintended variations in initial solid mixing dynamics or localized powder caking, which may have temporarily insulated the internal temperature probe tip during the earliest phase of the run.

Regarding reaction temperatures during the Diels–Alder step, the implementation of active cooling (Table 2, entry 5) demonstrated that the reaction remains effective even at substantially lower temperatures. In this experiment, the temperature was maintained between 11–14 °C compared to elevated temperatures (31–35 °C) for the bead-milling experiment without active cooling of the reaction chamber (Table 2, entry 4) and even higher temperatures of up to 53 °C due to friction heating in the ball-milling protocol with 30 minutes of milling time (Table 2, entry 2). In this context, and in line with the discussion in our previous work, where temperature effects could only be inferred indirectly due to the inherent coupling of milling frequency, mechanical impact, and frictional heating, the present results suggest that the Diels–Alder step is not critically dependent on elevated temperatures arising from frictional heating.

Notably, despite variations in operating temperatures across all experiments, the Diels–Alder cycloaddition always proceeded with exclusive *endo*-diastereoselectivity. Inspection of the crude ^1^H-NMR spectra for all runs revealed no detectable trace of characteristic signals to the alternative *exo*-cycloadduct (see SI).

### Wittig olefination

Following the successful demonstration of the one-pot sequence, we were further interested in the performance of the Wittig olefination under bead-milling conditions alone (details on the process steps and parameters are provided in the SI, Table S4). Under the optimized conditions (159 g beads, 5350 rpm), the olefination proceeded smoothly, affording the desired conjugated diene 2 in a good yield of 67% upon isolation within 10 minutes. This experiment corresponds to an approximately 60-fold scale-up relative to the reported ball-milling procedure (see Table 3),^26^ and confirms that bead milling provides sufficient mechanical activation to promote the Wittig reaction under solvent-free conditions.

**Table 3:** Comparison of the scale-up experiments under bead-milling conditions to the previously reported ball-milling approach^26^ of the Wittig olefination of aldehyde 1^,^^a^

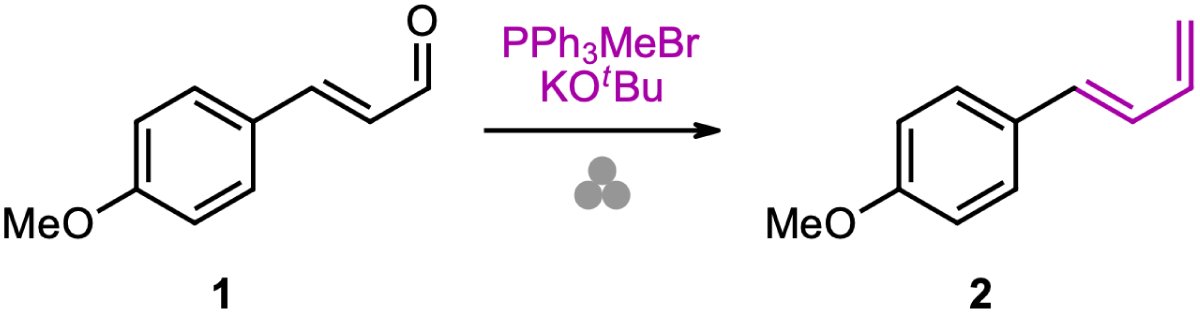							
Entry	Device	Frequency/tip speed [Hz/rpm]	Scale abs. (rel.) [mmol]	Time total [min]	Temp in/out^b^ [°C/°C]	Theoretical yield of 4 [g]	Isolated yield of 4 [g/%]
1^b^	Ball mill	36/—	0.5 (×1)	0.5	27.5/32.5	0.08	0.07/84
2^b^	Ball mill	36/—	2.0 (×4)	0.5	—/—	0.32	0.26/81
3^c^	DYNO®-MILL	—/5350	30.2 (×60)	10	24.4/22.8	4.84	3.27/67

a Temperature of the reaction mixture at the beginning and end of the Wittig olefination; for ball milling experiments, temperature measurements were performed immediately after the milling process with an infrared thermometer, focusing the sensor on the stainless-steel ball in the milling vessel.^26^ For bead milling experiments (DYNO®-MILL), process temperatures were monitored with a temperature probe inserted horizontally through the outlet lid to measure the temperature of the reaction mixture/bead bed.

b Aldehyde 1 (1.0 equiv.), PPh_3_MeBr (1.2 equiv.) and KO^*t*^Bu (1.4 equiv.).

c Reaction was performed with altered stoichiometric amounts using aldehyde 1 (1.0 equiv.), PPh_3_MeBr (1.4 equiv.) and KO^*t*^Bu (1.2 equiv.), which was demonstrated to proceed with the same efficiency under ball-milling conditions.^25^

Notably, in an initial attempt, prior cooling of the milling chamber resulted in condensation of atmospheric moisture on the internal surfaces. This moisture caused a marked decrease in reaction performance, which we attribute to the rapid hydrolysis of the moisture-sensitive KO^*t*^Bu base. Thorough drying of the equipment with paper towels restored reproducible and efficient olefination under otherwise identical conditions.

## Conclusion

In summary, this study demonstrates that agitator bead mills have the potential to allow the rapid translation of established mechanochemical reaction concepts from laboratory-scale ball milling to preparative-scale operation. The one-pot Wittig–Diels–Alder sequence was successfully implemented in only three runs by sequentially adjusting the overall filling degree, accelerator speed, and bead-filling degree, while maintaining reasonable reaction times despite the substantially increased scale. Although the individual steps were not fully optimized, efficient conversions were achieved without fundamental changes to the reaction design and within only three working days.

Notably, the Diels–Alder step proceeded well even at lower reaction temperatures under bead-milling conditions, indicating that elevated temperatures arising from frictional heating are not required for a successful cycloaddition in this system. Overall, these findings highlight agitator bead mills as practical devices for the scale-up of mechanochemical transformations, bridging laboratory-scale mechanochemical studies and preparative synthesis with minimal process adaptation.

## Conflicts of interest

P. M. C. R. and K. B. are current employees of Willy A. Bachofen AG (WAB®), which manufactures the bead mill equipment evaluated in this manuscript. The experiments were partially conducted at WAB facilities with the assistance of WAB staff. The remaining authors declare no conflicts of interest.

## Supplementary Material

MR-003-D6MR00016A-s001

## Data Availability

The data supporting this article are available within the paper and its supplementary information (SI). Supplementary information: specifications on experimental set-up, detailed experimental procedures, analysis, and NMR spectra of products. See DOI: https://doi.org/10.1039/d6mr00016a.
