# Supplementary material for: From ball-milling to bead-milling technology: rapid optimization and scale-up of a one-pot Wittig olefination–Diels–Alder reaction sequence using an agitator bead mill
Source: RSC Mechanochem. 2026 Jul 7. Online ahead of print. doi: 10.1039/d6mr00016a (PMC13359023; doi:10.1039/d6mr00016a)

Supplementary Information (SI) for RSC Mechanochemistry.
This journal is © The Royal Society of Chemistry 2026

From ball-milling to bead-milling technology: Rapid optimization and scale-up of a one-pot Wittig olefination–Diels-Alder reaction sequence using an agitator bead mill

Supplementary Information

Nina Biedermann,[a] Johanna Templ,[a] Kenneth Banderob,[b] Philippe M. C. Roth[b] and Michael Schnürch*[a]

[a] Institute of Applied Synthetic Chemistry, TU Wien
Getreidemarkt 9/163, 1060 Vienna, Austria
E-mail: michael.schnuerch@tuwien.ac.at

[b] Willy A. Bachofen AG
Junkermattstrasse 11, 4132 Muttenz, Switzerland

Table of Contents

1 General Information....SI-3

1.1 General Experimental Details....SI-3

1.2 DYNO®-MILL RESEARCH LAB....SI-4

2 Experimental Procedures....SI-5

2.1 Initial Optimization Experiment of the One-Pot Wittig Olefination–Diels-Alder Reaction Sequence under Ambient Conditions....SI-5

2.2 One-Pot Wittig Olefination–Diels-Alder Reaction Sequence under Slightly Optimized Reaction Parameters and Ambient Conditions....SI-5

2.3 One-Pot Wittig Olefination–Diels-Alder Reaction Sequence with Active Cooling in the Diels-Alder Step....SI-6

2.4 Wittig Olefination of Aldehyde 1....SI-8

2.5 Details on Process Steps and Parameters....SI-9

3 Analytical Investigations....SI-11

3.1 Crude Reaction Mixture Analysis....SI-11

3.2 HPLC-MS Analysis of Product 4 from Key Experiments....SI-15

4 Appendix....SI-16

4.1 References....SI-16

4.2 NMR Spectra of Products....SI-16

1 General Information

1.1 General Experimental Details

All chemicals were purchased from commercial suppliers with purities >95% and used without further purification. To ensure that potassium tert-butoxide (KO^tBu) remained dry and active, the stock container was flushed with argon after each use and stored tightly sealed. Weighing was performed under ambient atmosphere immediately prior to use, and the base was promptly charged into the grinding chamber to minimize exposure to atmospheric moisture. No further precautions were required

Reactions were conducted in a DYNO®-MILL RESEARCH LAB from Willy A. Bachofen AG (WAB-GROUP®) in batch mode (for technical details see Chapter 1.2). Grinding beads with a diameter of 8 mm made of yttria-stabilized zirconia (ZrO_2/Y_2O_3) were used, and the bead filling volume is indicated as ϕ (e.g., 30% filling volume corresponds to ϕ = 0.3).

Column chromatography was performed using standard manual glass columns packed with Merck silica gel 60 (40–63 µm) and light petroleum (LP)/ethylacetate (EtOAc). Thin-layer chromatography (TLC) analysis was performed on aluminum-backed unmodified Merck silica gel 60 F_{245} plates. Visualization was achieved under UV irradiation or by heat staining with a potassium permanganate ($KMnO_4$) solution.

^{1}H NMR and ^{13}C NMR spectra were recorded on a Bruker Avance UltraShield 400 MHz or Avance NEO 400 MHz spectrometer at ambient temperature. Processing of the data was performed with standard software, and all spectra were calibrated to the solvent residual peak, unless stated otherwise. Chemical Shifts (δ) are reported in ppm and multiplicities are assigned as s (singlet), d (doublet), t (triplet), q (quartet), m (multiplet), and combinations thereof, coupling constants (*J*) are reported in Hertz (Hz).

HPLC-MS analysis was performed on a Nexera LC-40XR system (Shimadzu, Kyoto, Japan) comprising LC-40D XR pumps, a SIL-40C XR autosampler, a CTO-40C column oven, CBH-40 system controller, and a DGU-405 degasser. Detection was carried out using an SPD-M40 photodiode array detector, an ELSD LTIII evaporative light scattering detector, and an LCMS-2050 mass spectrometer (DUIS). Separations were achieved on an XSelect CSH C18 XP column (130 Å, 3.5 µm, 50 × 3.0 mm, Waters, Milford, MA, USA) at 40 °C with a flow rate of 1.3 mL min^{-1}. The mobile phases consisted of UHPLC-grade water containing 0.1% (v/v) formic acid (A) and acetonitrile (B). The following gradient was used: 0–0.15 min, 5% B; 0.15–2.5 min, linear gradient to 98% B; 2.5–2.8 min, 98% B; 2.8–3.0 min, 5% B.

Melting points were recorded using a BÜCHI Melting Point B545 with a 40%/90% threshold and a heating rate of 1.0 °C/min.

1.2 DYNO®-MILL RESEARCH LAB

Reactions were conducted in a DYNO®-MILL RESEARCH LAB from Willy A. Bachofen AG (WAB-GROUP®) (Figure S-1). The mill features the patented DYNO®-ACCELERATOR made of stainless steel, which is driven by a motor at tip speeds ranging from 6–16 m/s (2200–6000 rpm), and a cylindrical grinding chamber (V = 80 mL) made of hardened stainless steel. In general, the system comprises a feed funnel and a recirculation loop that enables circulation of the reaction mixture, as depicted in Figure S-1 (left). For batch operation, these components can be removed and the outlet sealed with a batch cap made of hardened stainless steel (1.4404), resulting in the configuration shown in Figure S-1 (right). The mill is equipped with three independent cooling circuits: 1) water cooling for the motor, 2) cooling for the mechanical seal with a water/glycol mixture (1:1), and 3) a water/glycol circuit for the reactive grinding chamber. Temperature control of the reactive chamber is achieved using an external thermostat, and the reaction temperature was monitored via a temperature probe installed at the batch cap, inserted horizontally to measure the temperature of the reaction mixture/bead bed.

In this work, the DYNO®-MILL RESEARCH LAB was used in a batch-mode setup as depicted in Figure S-1 (right).

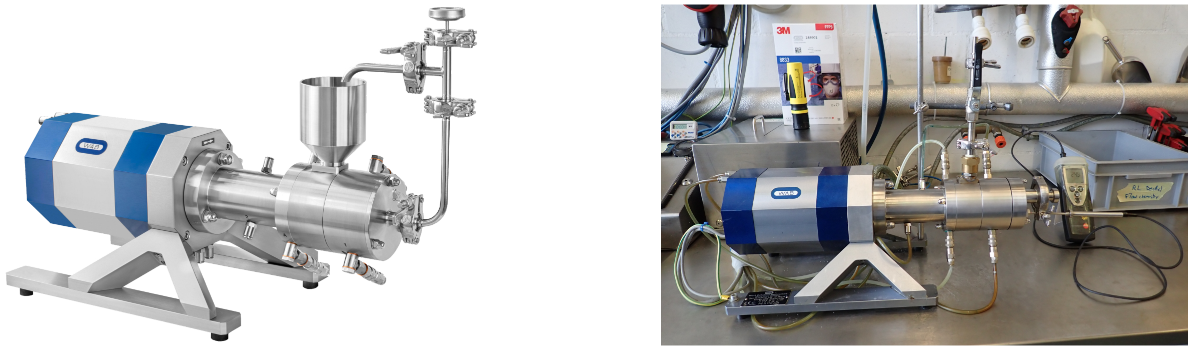

Figure S-1. DYNO®-MILL RESEARCH LAB; left: continuous-operation setup featuring a feed funnel and recirculation loop; right: batch-mode setup used in this work.

2 Experimental Procedures

2.1 Initial Optimization Experiment of the One-Pot Wittig Olefination–Diels-Alder Reaction Sequence under Ambient Conditions

The grinding chamber of the DYNO®-MILL RESEARCH LAB (V = 80 mL) was filled with ZrO_2/Y_2O_3 beads (Ø = 0.8 mm, m = 91 g, ϕ = 0.30), and the motor cooling circuit was set to 200 L/h with an inlet temperature of 19.1 °C. (*E*)-4-methoxycinnamaldehyde (**1**) (5.00 g, 30.2 mmol, 1.00 equiv.), methyltriphenylphosphonium bromide (15.4 g, 42.3 mmol, 1.40 equiv.), and potassium *tert*-butoxide (4.07 g, 36.2 mmol, 1.20 equiv.) were added to the container, and grinding was initially performed at a tip speed of 4 m/s for 5 minutes. Reaction progress was monitored via TLC (micro work-up with sat. aq. NH_4Cl and EtOAc), which showed incomplete conversion. The grinding process was then prolonged by 5 minutes at a higher tip speed of 14 m/s. Since TLC still indicated low conversion, additional zirconia beads (Ø = 0.8 mm; total: m = 159 g; ϕ = 0.525) were added to improve grinding efficiency. After an additional 10 minutes, TLC indicated complete conversion of the aldehyde. Solid NH_4Cl (1.62 g, 30.2 mmol, 1.00 equiv.) was added, and grinding was continued at 14 m/s for 5 minutes to quench remaining base. Subsequently, N-phenylmaleimide (3) (5.34 g, 30.2 mmol, 1.00 equiv.) was added, and the mixture was ground for 30 minutes at 14 m/s under cooling. For reaction monitoring, a small sample of the crude mixture was added to a solution of maleimide (100 mg) in EtOAc (1 mL), and TLC was performed. Due to the detection of minor quench product, the Diels-Alder step was prolonged by 15 minutes until TLC confirmed complete conversion of the intermediate diene **2** during grinding.

The mill was disassembled, the crude mixture was washed out with EtOAc and water, and the beads were recovered via filtration through a sieve. The phases were separated, the organic phase was washed with water (2×), and the combined aq. phase was extracted with EtOAc (2×). The combined EtOAc phase was finally washed with sat. aq. NH_4Cl, dried over anhydrous Na_2SO_4 and evaporated.

The crude product was purified via flash column chromatography (dry load with celite, silica, LP/EtOAc mixture) to obtain a yellow solid. This was triturated with cold Et_2O/EtOAc (approx. 1:1), resulting in 4.73 g (48%) of *rel*-(3a*R*,4*R*,7a*S*)-4-(4-methoxyphenyl)-2-phenyl-3a,4,7,7a-tetrahydro-2*H*-isoindole-1,3-dione (**4**) as a colorless to slightly yellow solid.

Spectral data is in accordance with previous work.[1]

2.2 One-Pot Wittig Olefination–Diels-Alder Reaction Sequence under Slightly Optimized Reaction Parameters and Ambient Conditions

The grinding chamber of the DYNO®-MILL RESEARCH LAB (V = 80 mL) was filled with ZrO_2/Y_2O_3 beads (Ø = 0.8 mm, m = 91 g, ϕ = 0.30) and the reactants (*E*)-4-methoxycinnamaldehyde (**1**) (5.00 g, 30.2 mmol, 1.00 equiv.), methyltriphenylphosphonium bromide (15.4 g, 42.3 mmol, 1.40 equiv.), and potassium *tert*-butoxide (4.07 g, 36.2 mmol, 1.20 equiv.) in layers of beads–reactants–beads. The motor cooling circuit was set to 200 L/h with an inlet temperature of 14.2 °C (Fluctuations in the motor cooling water temperature reflect daily variations in the facility's central cooling line; note that this circuit cools only the drive motor and does not affect the reaction chamber), and grinding was performed at 14 m/s for 5 minutes. Reaction progress was monitored via TLC (micro work-up with sat. aq. NH_4Cl and EtOAc), showing complete conversion. Solid NH_4Cl (1.62 g, 30.2 mmol, 1.00 equiv.) was added, and grinding was continued at 14 m/s for 5 minutes to quench remaining base.
Subsequently, *N*-phenylmaleimide (**3**) (5.34 g, 30.2 mmol, 1.00 equiv.) was added, and grinding was performed for 30 minutes at 14 m/s. For reaction monitoring, a small sample of the crude mixture was added to a solution of maleimide (100 mg) in EtOAc (1 mL), and TLC was performed.

The mill was disassembled, the crude mixture was washed out with EtOAc and water, and the beads were recovered via filtration through a sieve. The phases were separated, the organic phase was washed with water (2×), and the combined aq. phase was extracted with EtOAc (2×). The combined EtOAc phase was finally washed with sat. aq. NH_4Cl, dried over anhydrous Na_2SO_4, and evaporated.

Figure S-2 shows photographs of the used agitator bead mill and the reaction mixture at different steps in the process.

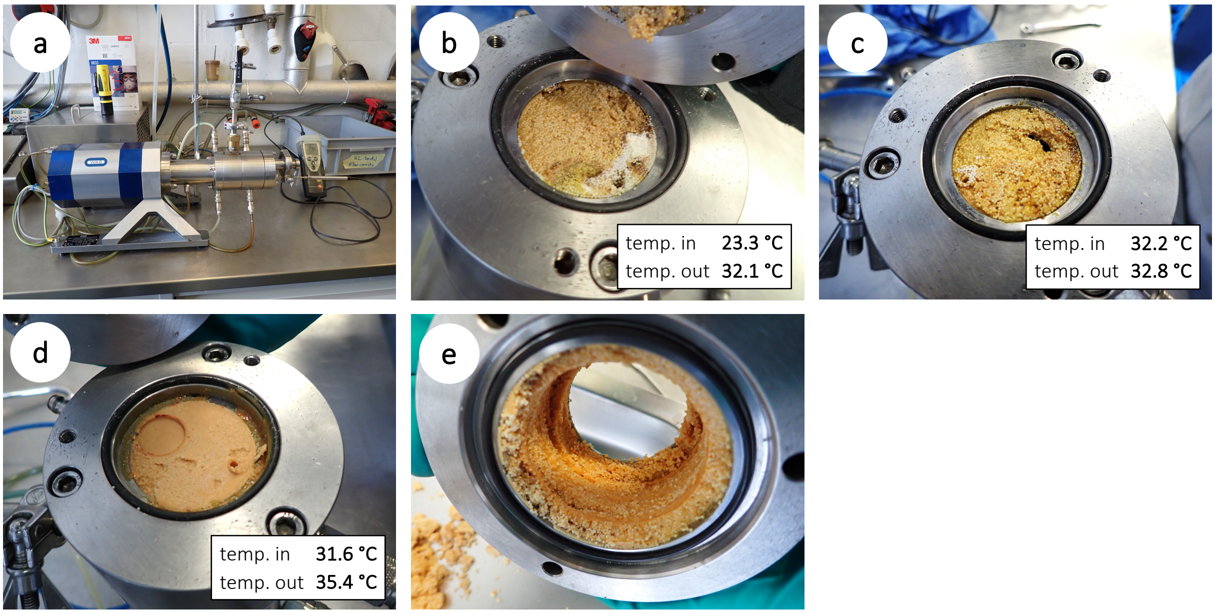

Figure S-2. Pictures of the one-pot Wittig olefination–Diels-Alder reaction sequence of **1** in a DYNO®-MILL RESEARCH LAB under ambient, optimized conditions. a) DYNO®-MILL RESEARCH LAB set-up used in this work; b) reaction mixture after the Wittig olefination step with aldehyde **1**, PPh_3MeBr, KO^{t}Bu and ZrO_2/Y_2O_3 grinding beads (Ø = 0.8 mm) before the addition of NH_4Cl; c) reaction mixture after the quenching step with NH_4Cl; e) reaction mixture after the Diels-Alder step; f) final crude product bed after disassembling the mill. Recorded internal temperatures of the reaction mixture at the beginning and end of each process step are indicated in pictures c, d, and e as *temp. in* and *temp. out*, respectively, to track the system's thermal profile.

The crude product was purified via flash column chromatography (dry load with celite, silica, LP/EtOAc mixture) to obtain a yellow solid. This was triturated with cold Et_2O/EtOAc (approx. 1:1), resulting in 6.72 g (67%) of *rel*-(3a*R*,4*R*,7a*S*)-4-(4-methoxyphenyl)-2-phenyl-3a,4,7,7a-tetrahydro-2*H*-isoindole-1,3-dione (**4**) as a colorless to slightly yellow solid (m.p. 146.4-147.6 °C).

Spectral data is in accordance with previous work.[1]

^{1}H NMR (400 MHz, $CDCl_3$) δ 7.34 – 7.24 (m, 3H), 7.15 – 7.07 (m, 2H), 6.87 – 6.78 (m, 2H), 6.70 – 6.60 (m, 2H), 6.20 – 6.07 (m, 2H), 3.95 – 3.89 (m, 1H), 3.76 (s, 3H), 3.43 (dd, *J* = 8.9, 7.2 Hz, 1H), 3.39 – 3.30 (m, 1H), 3.04 – 2.94 (m, 1H), 2.63 – 2.51 (m, 1H).

^{13}C NMR (101 MHz, $CDCl_3$) δ 178.8, 176.9, 159.2, 131.7, 130.5, 130.2, 129.4, 128.9, 128.5, 126.8, 126.4, 114.1, 55.5, 44.9, 40.2, 38.0, 21.7.

2.3 One-Pot Wittig Olefination–Diels-Alder Reaction Sequence with Active Cooling in the Diels-Alder Step

This experiment was performed with active cooling of the reactive chamber in the Diels-Alder step using an additional cooling system (water/glycol mixture).

The grinding chamber of the DYNO®-MILL RESEARCH LAB (V = 80 mL) was filled with ZrO_2/Y_2O_3 beads (Ø = 0.8 mm, m = 159 g, ϕ = 0.525), and the motor cooling circuit was set to 200 L/h with an inlet temperature of 18.4 °C. (*E*)-4-methoxycinnamaldehyde (**1**) (5.00 g, 30.2 mmol, 1.00 equiv.), methyltriphenylphosphonium bromide (15.4 g, 42.3 mmol, 1.40 equiv.), and potassium *tert*-butoxide (4.07 g, 36.2 mmol, 1.20 equiv.) were added to the container, and grinding was performed with a tip speed of 14 m/s for 10 minutes. Reaction progress was monitored via TLC (micro work-up with sat. aq. NH_4Cl and EtOAc), and grinding was continued for an additional 5 minutes, until TLC confirmed complete conversion.

Subsequently, the cooling system for the reactive chamber was switched on, solid NH_4Cl (30.2 mmol, 1.62 g, 1.00 equiv.) was added, and grinding was continued at 14 m/s for 5 minutes to quench remaining base. To allow the reaction mixture to cool down, this step was extended by 10 minutes at a lower tip speed of 4 m/s to reach a crude product temperature of 10.2 °C. Then, *N*-phenylmaleimide (**3**) (5.34 g, 30.2 mmol, 1.00 equiv.) was added, and grinding was performed for 30 minutes at 14 m/s while cooling. For reaction monitoring, a small sample of the crude mixture was added to a solution of maleimide (100 mg) in EtOAc (1 mL), and TLC was performed, confirming complete conversion of the intermediate diene **2** in the grinding process.

The mill was disassembled, the crude mixture was washed out with EtOAc and water, and the beads were recovered via filtration through a sieve. The phases were separated, the organic phase was washed with water (2×), and the combined aq. phase was extracted with EtOAc (2×). The combined EtOAc phase was finally washed with sat. aq. NH_4Cl, dried over anhydrous Na_2SO_4 and evaporated.

Figure S-3 shows photographs of the used agitator bead mill and the reaction mixture at different steps in the process.

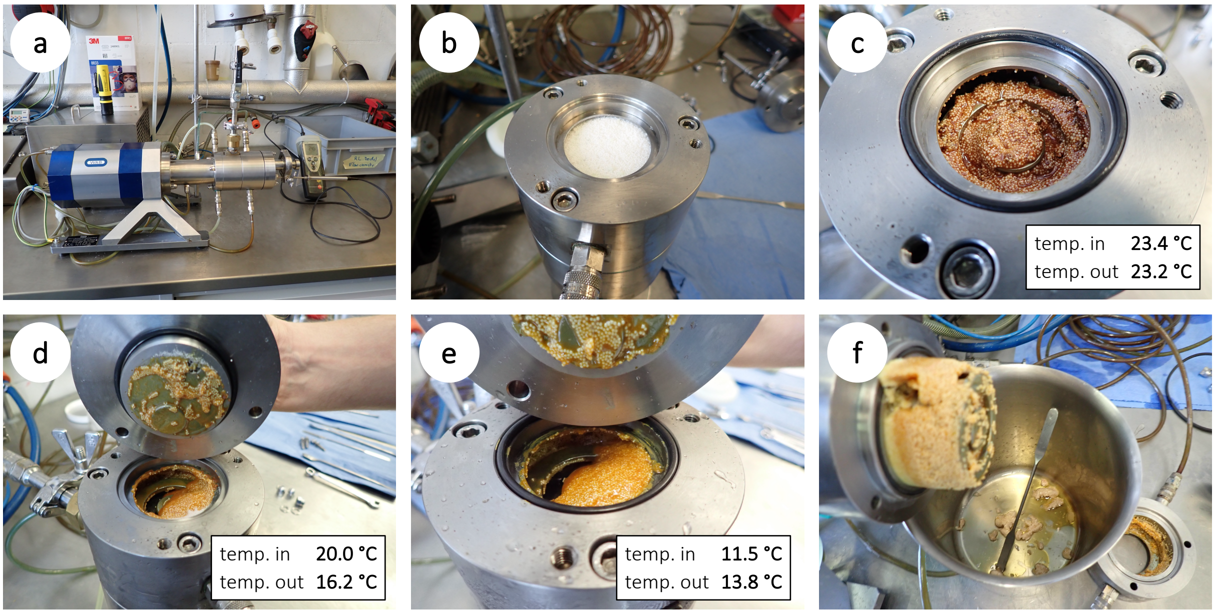

Figure S-3. Pictures of the one-pot Wittig olefination–Diels-Alder reaction sequence of **1** in a DYNO®-MILL RESEARCH LAB with active cooling of the reactive chamber. a) DYNO®-MILL RESEARCH LAB set-up used in this work; b) initial solid mixture – grinding chamber filled with ZrO_2/Y_2O_3 grinding beads (Ø = 0.8 mm), aldehyde **1**, PPh_3MeBr and KO^tBu; c) reaction mixture after the Wittig olefination step before the addition of NH_4Cl; d) reaction mixture after the quenching step with NH_4Cl, where the cooling of the grinding chamber has already been engaged; e) reaction mixture after the Diels-Alder step under active cooling; f) final crude product bed after disassembling the mill, and scraping and washing down of the reaction mixture from the accelerator. Recorded internal temperatures of the reaction mixture at the beginning and end of each process step are indicated in pictures c, d, and e as *temp. in* and *temp. out*, respectively, to track the system's thermal profile.

The crude product was further purified via flash column chromatography (dry load with celite, silica, LP/EtOAc mixture) to obtain 6.12 g of a yellowish solid. This was triturated with cold Et_2O/EtOAc (approx. 1:1), resulting in 5.99 g (60%) of *rel*-(3a*R*,4*R*,7a*S*)-4-(4-methoxyphenyl)-2-phenyl-3a,4,7,7a-tetrahydro-2*H*-isoindole-1,3-dione (**4**) as a colorless to slightly yellow solid (m.p. 146.7-148.3 °C).

2.4 Wittig Olefination of Aldehyde 1

The grinding chamber of the DYNO®-MILL RESEARCH LAB (V = 80 mL) was filled with ZrO_2/Y_2O_3 beads (Ø = 0.8 mm, m = 159 g, ϕ = 0.525), and the motor cooling circuit was set to 200 L/h with an inlet temperature of 19.6 °C. (*E*)-4-methoxycinnamaldehyde (**1**) (5.00 g, 30.2 mmol, 1.00 equiv.), methyltriphenylphosphonium bromide (15.4 g, 42.3 mmol, 1.40 equiv.), and potassium *tert*-butoxide (4.07 g, 36.2 mmol, 1.20 equiv.) were added to the container, and grinding was performed with a tip speed of 14 m/s for 10 minutes. After confirmation of complete conversion via TLC (micro work-up with sat. aq. NH_4Cl and EtOAc), solid NH_4Cl (1.62 g, 30.2 mmol, 1.00 equiv.) was added, and grinding was continued at 14 m/s for 8 minutes to quench remaining base.

The mill was disassembled, the crude mixture was washed out with EtOAc and water, and the beads were recovered via filtration through a sieve. The phases were separated, the organic phase was washed with water (2×), and the combined aq. phase was extracted with EtOAc (2×). The combined EtOAc phase was finally washed with sat. aq. NH_4Cl, dried over anhydrous Na_2SO_4 and evaporated.

The crude product was purified via flash column chromatography (dry load with celite, silica, LP/EtOAc mixture) to obtain 3.27 g (67% yield) of 4-[(1*E*)-buta-1,3-dien-1-yl]-1-methoxybenzene (**2**) as a colorless solid.

Spectral data is in accordance with previous work.[1]

^{1}H NMR (400 MHz, $CDCl_3$) δ 7.38 – 7.32 (m, 2H), 6.91 – 6.82 (m, 2H), 6.72 – 6.63 (m, 1H), 6.57 – 6.43 (m, 2H), 5.32 – 5.26 (m, 1H), 5.15 – 5.10 (m, 1H), 3.82 (s, 3H).

^{13}C NMR (101 MHz, $CDCl_3$) δ 159.4, 137.5, 132.5, 130.1, 127.8, 116.6, 114.2, 55.4.

2.5 Details on Process Steps and Parameters

The following Tables S-1 to S-4 show the process steps and parameters for each reported experiment.

For clarification on the table parameters: *Time total* refers to the cumulative process duration, while *time step* characterizes the specific runtime of an individual operational step. *Product temp.* represents the internal temperature of the reaction mixture/bead bed measured via the temperature probe installed at the batch cap. *Cooling system temp.* indicates the temperature of the cooling liquid circulating through the jacket of the grinding chamber, whereas *cooling water flow* and *cooling water temp.* refer to the flow rate and temperature of the auxiliary water circuit for cooling of the motor unit.

Table S-1. Detailed process steps and parameters for the initial optimization run of the one-pot Wittig olefination–Diels-Alder reaction sequence at ambient conditions, described in Chapter 2.1.

**Experiment name:**	**Initial optimization run of the one-pot Wittig olefination–Diels-Alder reaction sequence at ambient conditions**
**Product name:**	4-(*p*-Methoxyphenyl)-2-phenyl-3a,4,7,7a-tetrahydro-2*H*-isoindole-1,3-dione (**4**)
**Reactants and reagents:**	(2*E*)-3-(4-Methoxyphenyl)prop-2-enal (**1**); PPh_3MeBr; KO^tBu; NH_4Cl; *N*-Phenylmaleimide (**3**)
**Mill type:**	DYNO®-MILL RESEARCH LAB
**Motor:**	1.1 kW; 3 × 400 V; 50 Hz; 4.0 A
**Inner cylinder:**	V = 80 mL; Hardened stainless steel
**Agitators:**	DYNO®-ACCELERATOR; Hardened chrome alloy
**Beads:**	Yttria-stabilized zirconia (ZrO_2/Y_2O_3); Ø = 0.8 mm; m = 159 g; φ = 0.525

Step	Time total	Time step	Bead filling	Tip speed	Product temp.	Product temp.	Cooling system temp.	Cooling system temp.	Cooling water flow	Cooling water temp.	Cooling water temp.	Comments
	[min]	[min]	[%]	[m/s]	Start [°C]	End [°C]	IN [°C]	OUT [°C]	[L/h]	IN [°C]	OUT [°C]	
**1**	5	5	30.0	10	24.2	25.2	/	/	200	19.1	21.1	Wittig reaction: aldehyde **1**, PPh_3MeBr, KO^tBu
**2**	10	5	30.0	14	22.8	21.7	/	/	200	19.1	22.4	Reaction incomplete; Grinding prolonged (+5) at higher tip speed
**3**	15	5	30.0	14	22.3	22.4	/	/	200	19.1	22.3	Reaction incomplete; Addition of beads (68 g) (total: 159 g, φ = 0.525); Grinding prolonged (+5)
**4**	20	5	52.5	14	22.1	21.8	/	/	200	19.1	22.4	Reaction incomplete; Grinding prolonged (+5)
**5**	25	5	52.5	14	21.6	22.6	/	/	200	19.1	22.7	Base quench: Addition of NH_4Cl
**6**	55	30	52.5	14	22.4	24.2	/	/	200	19.1	24.5	Base quench: Addition of NH_4Cl
**7**	70	15	52.5	14	23.3	22.5	/	/	200	19.1	24.6	Reaction incomplete; Grinding prolonged

Table S-2. Detailed process steps and parameters for the one-pot Wittig olefination–Diels-Alder reaction under optimized reaction parameters and ambient conditions, described in Chapter 2.2.

**Experiment name:**	**One-pot Wittig olefination–Diels-Alder reaction under optimized conditions**
**Product name:**	4-(*p*-Methoxyphenyl)-2-phenyl-3a,4,7,7a-tetrahydro-2*H*-isoindole-1,3-dione (**4**)
**Reactants and reagents:**	(2*E*)-3-(4-Methoxyphenyl)prop-2-enal (**1**); PPh_3MeBr; KO^tBu; NH_4Cl; *N*-Phenylmaleimide (**3**)
**Mill type:**	DYNO®-MILL RESEARCH LAB
**Motor:**	1.1 kW; 3 × 400 V; 50 Hz; 4.0 A
**Inner cylinder:**	V = 80 mL; Hardened stainless steel
**Agitators:**	DYNO®-ACCELERATOR; Hardened chrome alloy
**Beads:**	Yttria-stabilized zirconia (ZrO_2/Y_2O_3); Ø = 0.8 mm; m = 159 g; φ = 0.525

Step	Time total	Time step	Bead filling	Tip speed	Product temp.	Product temp.	Cooling system temp.	Cooling system temp.	Cooling water flow	Cooling water temp.	Cooling water temp.	Comments
	[min]	[min]	[%]	[m/s]	Start [°C]	End [°C]	IN [°C]	OUT [°C]	[L/h]	IN [°C]	OUT [°C]	
**1**	10	10	52.5	14	23.3	32.1	/	/	200	14.2	17.7	Wittig reaction: aldehyde **1**, PPh_3MeBr, KO^tBu
**2**	15	5	52.5	14	32.2	32.8	/	/	200	14.2	17.5	Base quench: Addition of NH_4Cl
**3**	45	30	52.5	14	31.6	35.4	/	/	200	14.2	18.7	Diels-Alder: Addition of *N*-phenylmaleimide (**3**)

Table S-3. Detailed process steps and parameters for the one-pot Wittig olefination–Diels-Alder reaction with active product cooling in the Diels-Alder step, described in Chapter 2.3.

**Experiment name:**	**One-pot Wittig olefination–Diels-Alder reaction sequence under optimized conditions with active product cooling**
**Product name:**	4-(*p*-Methoxyphenyl)-2-phenyl-3a,4,7,7a-tetrahydro-2*H*-isoindole-1,3-dione (**4**)
**Reactants and reagents:**	(2*E*)-3-(4-Methoxyphenyl)prop-2-enal (**1**); PPh_3MeBr; KO^{t}Bu; NH_4Cl; *N*-Phenylmaleimide (**3**)
**Mill type:**	DYNO®-MILL RESEARCH LAB
**Motor:**	1.1 kW; 3 × 400 V; 50 Hz; 4.0 A
**Inner cylinder:**	V = 80 mL; Hardened stainless steel
**Agitators:**	DYNO®-ACCELERATOR; Hardened chrome alloy
**Beads:**	Yttria-stabilized zirconia (ZrO_2/Y_2O_3); Ø = 0.8 mm; m = 159 g; φ = 0.525

**Step**	**Time total**	**Time step**	**Bead filling**	**Tip speed**	**Product temp.**	**Product temp.**	**Cooling system temp.**	**Cooling system temp.**	**Cooling water flow**	**Cooling water temp.**	**Cooling water temp.**	**Comments**
	[min]	[min]	[%]	[m/s]	Start [°C]	End [°C]	IN [°C]	OUT [°C]	[L/h]	IN [°C]	OUT [°C]	
**1**	10	10	52.5	14	23.4	23.2	/	/	200	18.4	21.6	Wittig reaction: aldehyde **1**, PPh_3MeBr, KO^{t}Bu
**2**	15	5	52.5	14	20.0	16.2	10.5	11.0	200	18.4	21.8	Base quench: Addition of NH_4Cl;<br>Cooling system switched on
**3**	25	10	52.5	4	12.3	10.2	7.0	7.0	200	18.4	21.5	Extended grinding for cooling of mixture
**4**	55	30	52.5	14	11.5	13.8	7.5	7.5	200	18.4	22.7	Diels-Alder: Addition of *N*-phenylmaleimide (**3**)

Table S-4. Detailed process steps and parameters for the Wittig olefination of aldehyde **1**, described in Chapter 2.4.

**Experiment name:**	**Wittig olefination of aldheyde 1**
**Product name:**	4-[(1*E*)-Buta-1,3-dien-1-yl]-1-methoxybenzene (**2**)
**Reactants and reagents:**	(2*E*)-3-(4-Methoxyphenyl)prop-2-enal (**1**); PPh_3MeBr; KO^{t}Bu; NH_4Cl
**Mill type:**	DYNO®-MILL RESEARCH LAB
**Motor:**	1.1 kW; 3 × 400 V; 50 Hz; 4.0 A
**Inner cylinder:**	V = 80 mL; Hardened stainless steel
**Agitators:**	DYNO®-ACCELERATOR; Hardened chrome alloy
**Beads:**	Yttria-stabilized zirconia (ZrO_2/Y_2O_3); Ø = 0.8 mm; m = 159 g; φ = 0.525

**Step**	**Time total**	**Time step**	**Bead filling**	**Tip speed**	**Product temp.**	**Product temp.**	**Cooling system temp.**	**Cooling system temp.**	**Cooling water flow**	**Cooling water temp.**	**Cooling water temp.**	**Comments**
	[min]	[min]	[%]	[m/s]	Start [°C]	End [°C]	IN [°C]	OUT [°C]	[L/h]	IN [°C]	OUT [°C]	
**1**	10	10	52.5	14	24.4	22.8	–	–	200	19.6	22.4	Wittig reaction: aldehyde **1**, PPh_3MeBr, KO^{t}Bu
**2**	15	5	52.5	14	22.8	22.1	–	–	200	19.6	22.1	Base quench: Addition of NH_4Cl
**3**	18	10	52.5	14	21.6	21.2	–	–	200	19.6	21.5	Grinding prolonged (+3)

3 Analytical Investigations

3.1 Crude Reaction Mixture Analysis

As described in our previous work,[1] the Diels-Alder reaction could, in principle, also proceed in solution during the work-up procedure. To confirm the conversion to product **4** in the bead-milling process, a small sample of the crude reaction mixture, taken directly from the grinding cylinder after the process, was added to a solution of maleimide (MI, 200 mg) in 10 mL of EtOAc. The aq. work-up procedure, including extraction with water and saturation. aq. NH_4Cl, and back extraction of the combined aq. phase with EtOAc once. The combined org. phase was dried over Na_2SO_4 and evaporated under reduced pressure. The residue was dissolved in $CDCl_3$, and ^{1}H-NMR was recorded.

For all bead-milling experiments, described in Table 2 (entries 3–5) of the manuscript, complete conversion to product **4** was confirmed based on the absence of signals for the quench-product (cycloaddition product with MI) in the recorded NMRs, which all showed mainly the desired product **4**, reagent-based impurities (e.g., triphenylphosphine oxide), and residual EtOAc next to MI. In addition, only the *endo*- and no *exo*-diastereomer was detected in all crude reaction mixtures.

The following Figure S-4 and Figure S-5 show the crude NMR spectrum of key crude mixtures after the aq. work-up with MI.

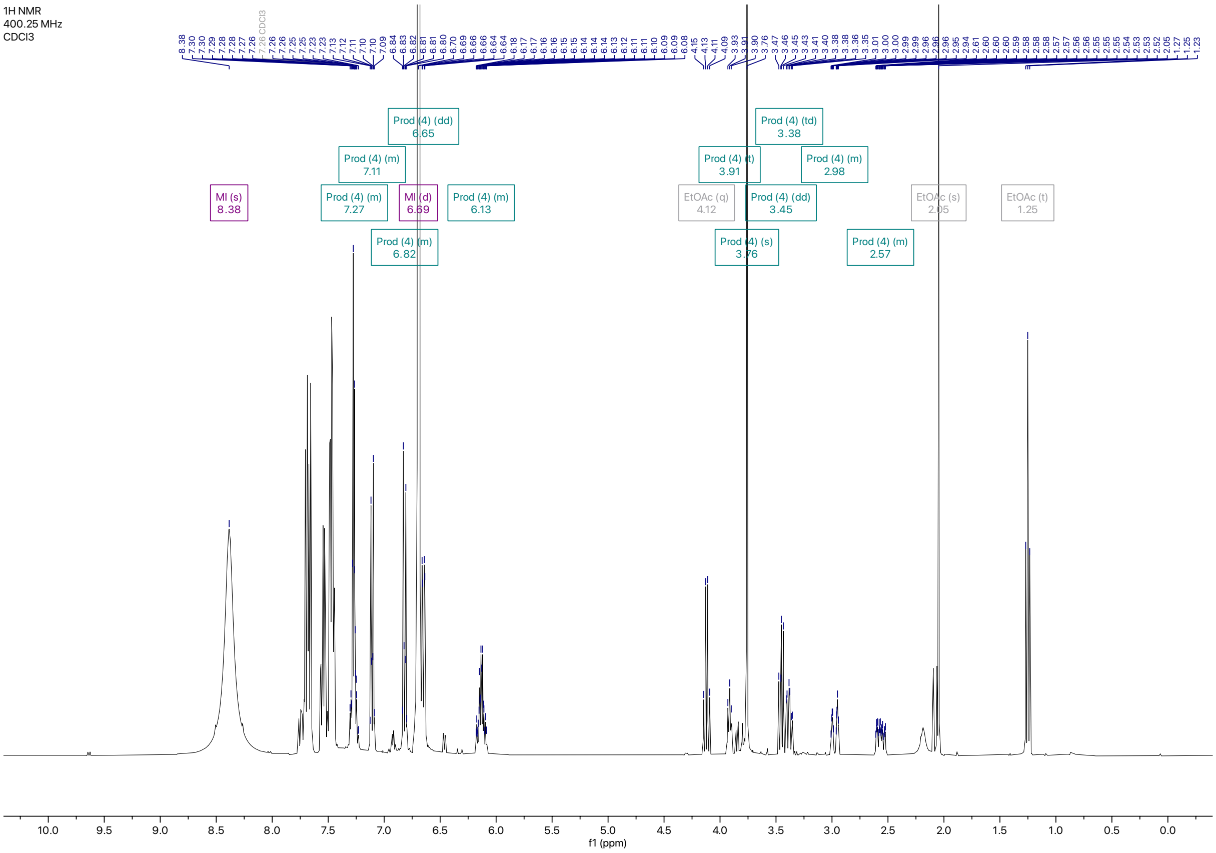

Figure S-4. ^{1}H-NMR (400 MHz, $CDCl_3$) of the crude reaction mixture from the optimized run (described in Chapter 2.2 and Table 2, entry 4), where the aq. work-up with maleimide (MI) was performed to confirm complete conversion in the bead-milling process.

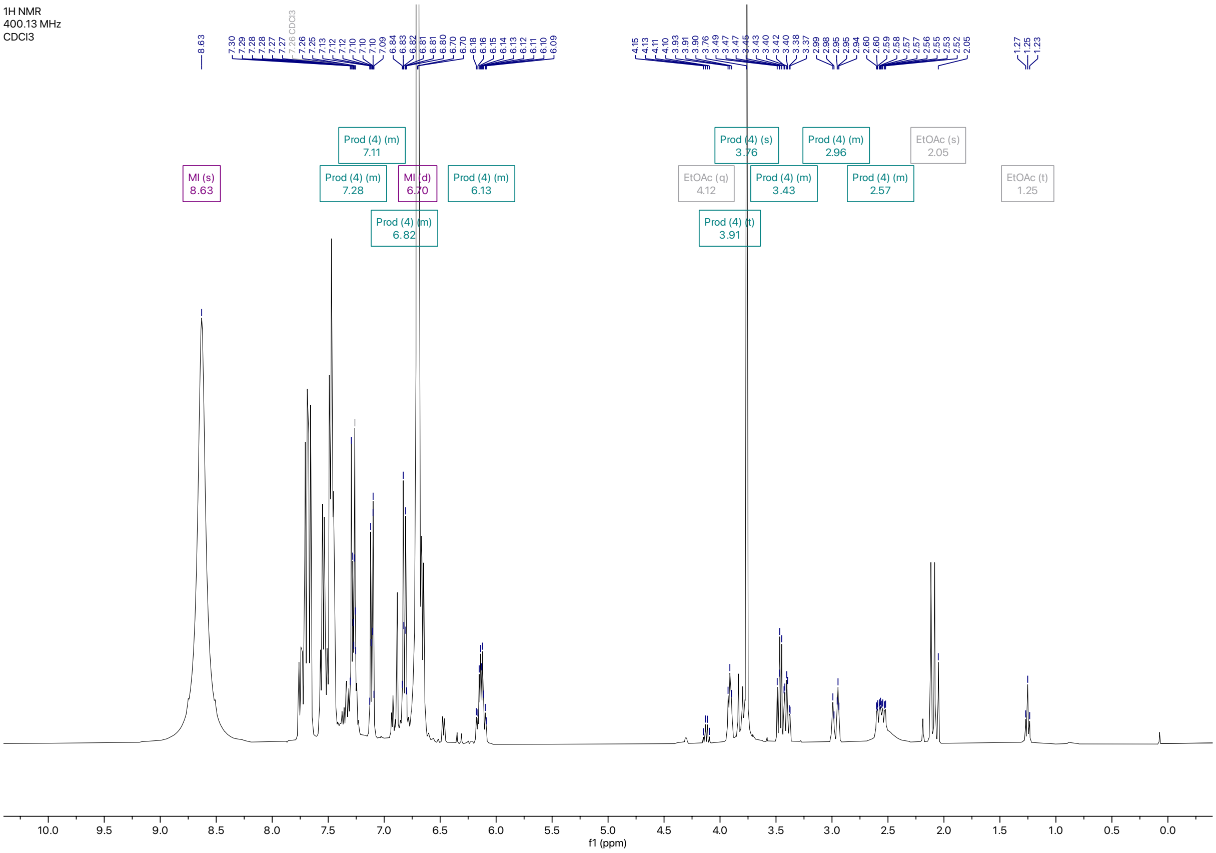

Figure S-5. ^{1}H-NMR (400 MHz, $CDCl_3$) of the crude reaction mixture from the optimized run with active product cooling in the Diels-Alder step (described in Chapter 2.3 and Table 2, entry 5), where the aq. work-up with maleimide (MI) was performed to confirm complete conversion in the bead-milling process.

In addition, we performed HPLC-MS analysis of crude reaction mixtures from the optimized run under ambient conditions (Chapter 2.2), after base quenching (Figure S-6) and the Diels-Alder step (Figure S-7). In both cases, aq. work-up was performed prior to the measurements to protect our HPLC-MS equipment. Because the MS spectra were recorded in the mass range of 200 to 2000 m/z, the low-molecular-weight intermediate product **2** was not detected under these conditions.

In the HPLC-MS chromatogram after the Wittig olefination and base quenching step (Figure S-6), a dominant peak corresponding to triphenylphosphine oxide (retention time: 1.840 min) was observed. Additionally, the crude chromatogram shows an unknown impurity with a retention time of 1.496 min, which exhibits a dominant mass signal at 217.064 m/z in the corresponding MS spectrum.

In addition to the peaks described for Figure S-6, the HPLC-MS chromatogram of the crude reaction mxiture after the complete one-pot sequence (Figure S-7) shows a dominant peak for final product **4** (retention time: 2.023 min), with a corresponding mass signal of m/z = 334.066, matching the expected [M+H]$^+$ ion.

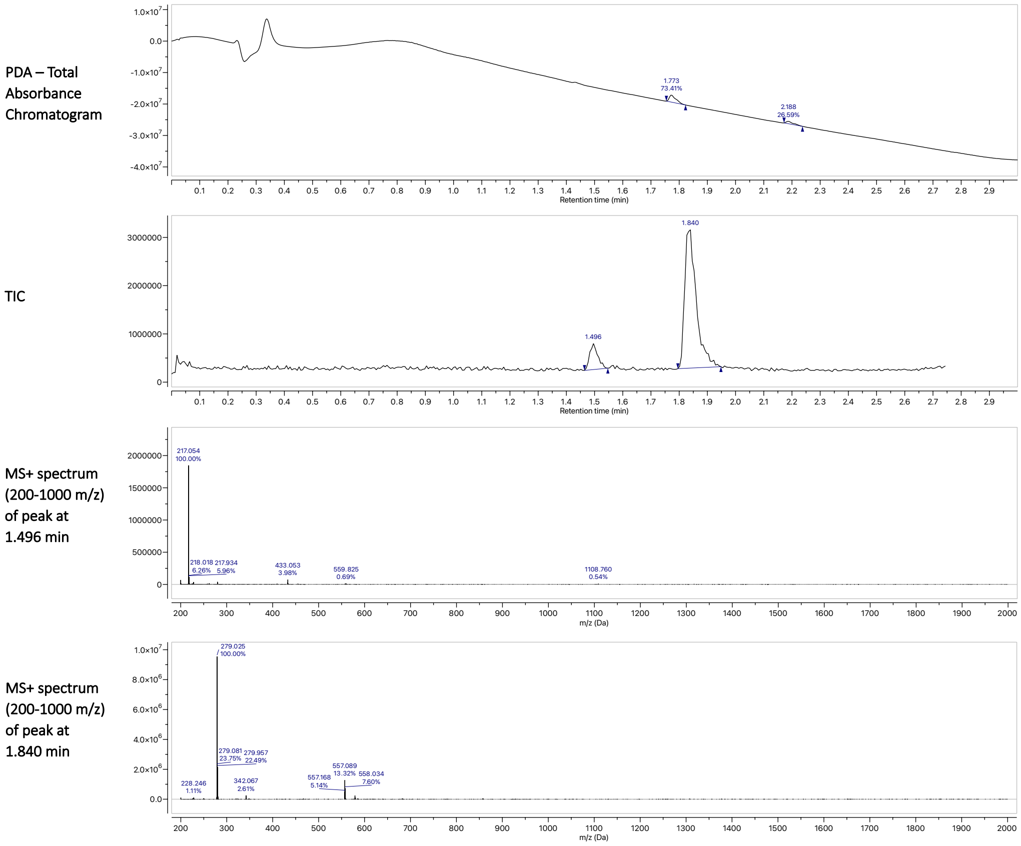

Figure S-6. HPLC-MS analysis of crude reaction mixture after the quenching step, after aq. work-up, obtained from the optimized run under ambient conditions (Chapter 2.2).

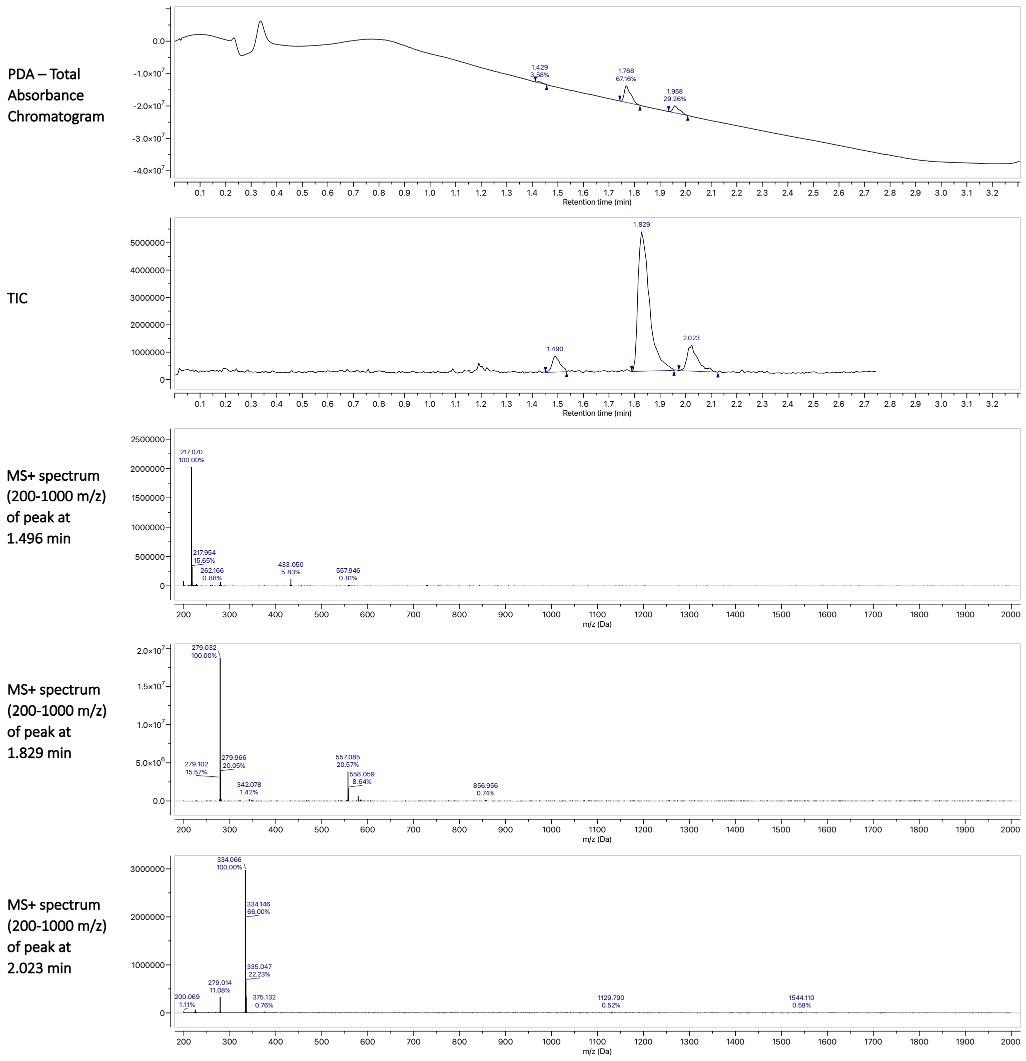

Figure S-7. HPLC-MS analysis of crude reaction mixture after the Diels-Alder step (whole one-pot sequence), after aq. work-up, obtained from the optimized run under ambient conditions (Chapter 2.2).

3.2 HPLC-MS Analysis of Product 4 from Key Experiments

HPLC-MS measurements were performed on the isolated scale-up products **4** to evaluate their chemical purity. For detailed instrument specifications and chromatographic method parameters, see Section 1.1 General Experimental Details.

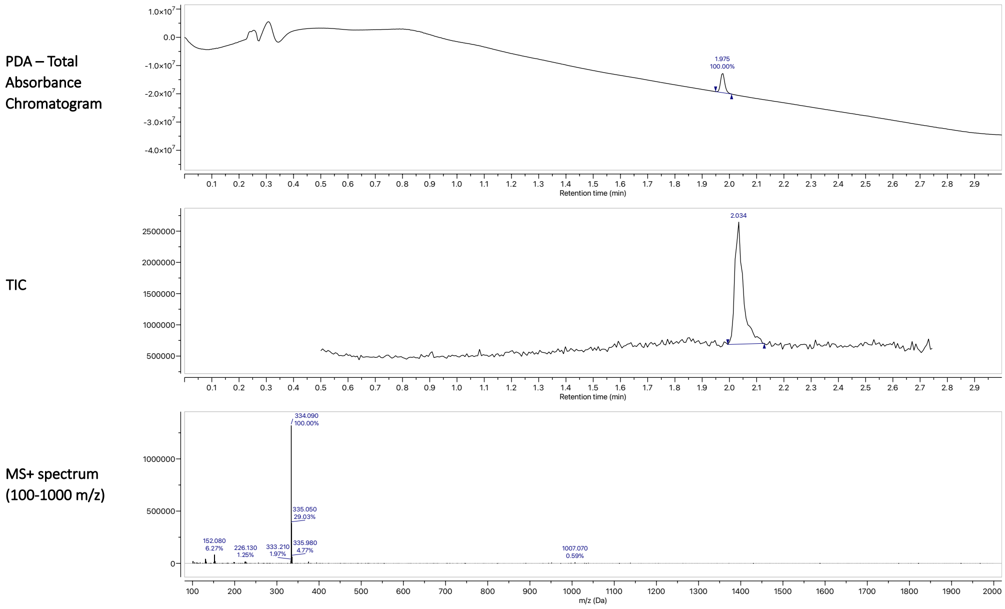

Figure S-8. HPLC-MS analysis of isolated product **4** from the optimized run under ambient conditions (Chapter 2.2).

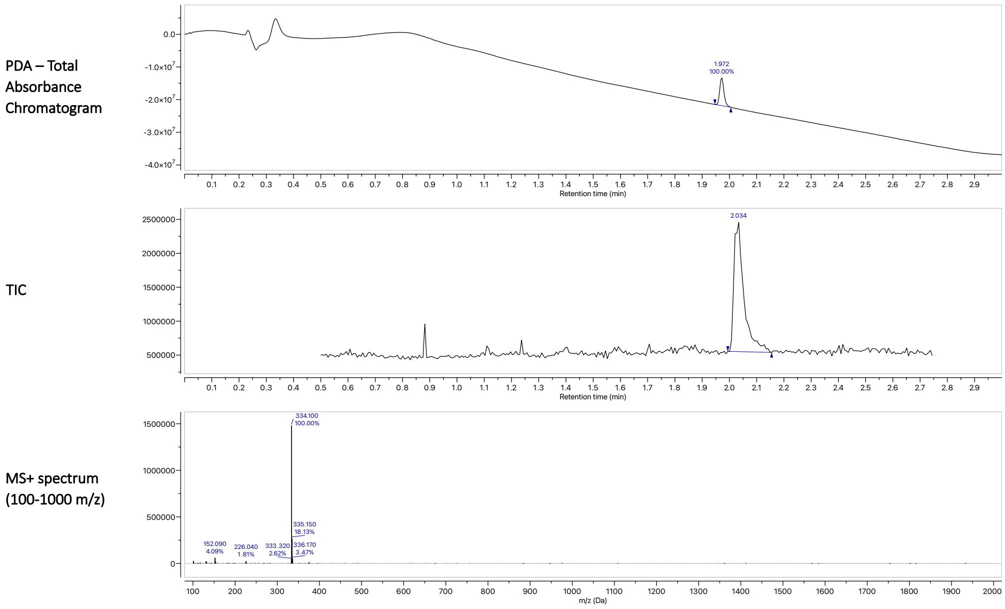

Figure S-9. HPLC-MS analysis of isolated product **4** from the optimized run with active cooling in the Diels-Alder step (Chapter 2.3).

4 Appendix

4.1 References

1. N. Biedermann and M. Schnürch, *RSC Mechanochem.*, 2025, **2**, 879-888.

4.2 NMR Spectra of Products

4-[(1*E*)-Buta-1,3-dien-1-yl]-1-methoxybenzene (**2**)

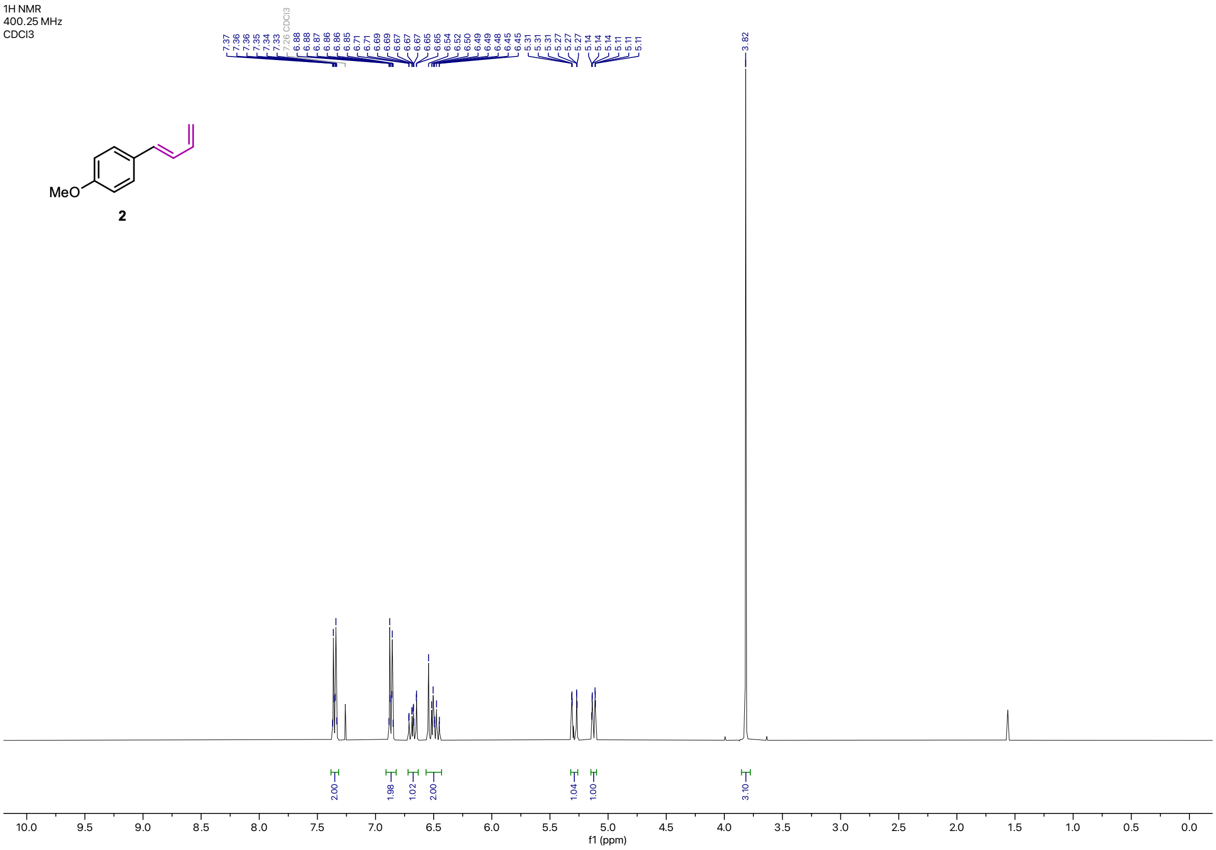

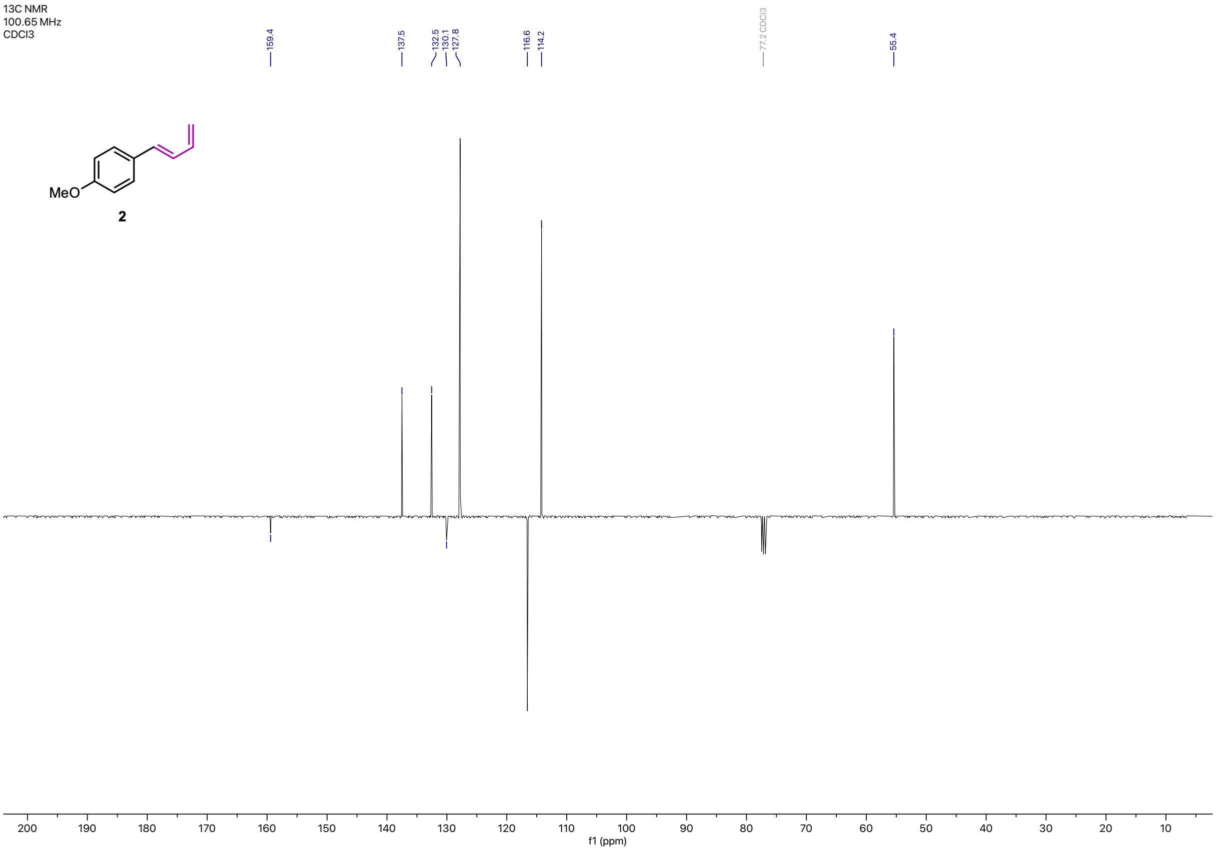

rel-(3a*R*,4*R*,7a*S*)-4-(4-Methoxyphenyl)-2-phenyl-3a,4,7,7a-tetrahydro-2*H*-isoindole-1,3-dione (**4**)

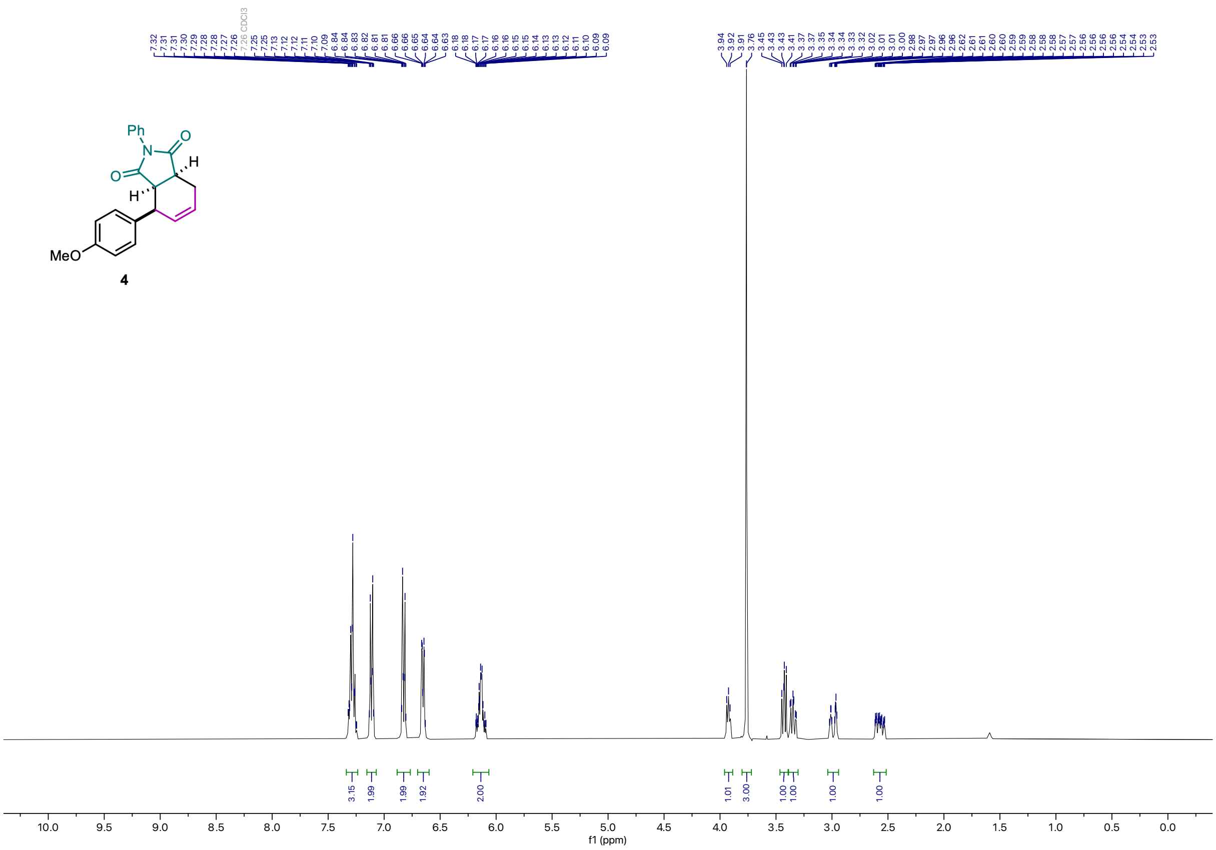

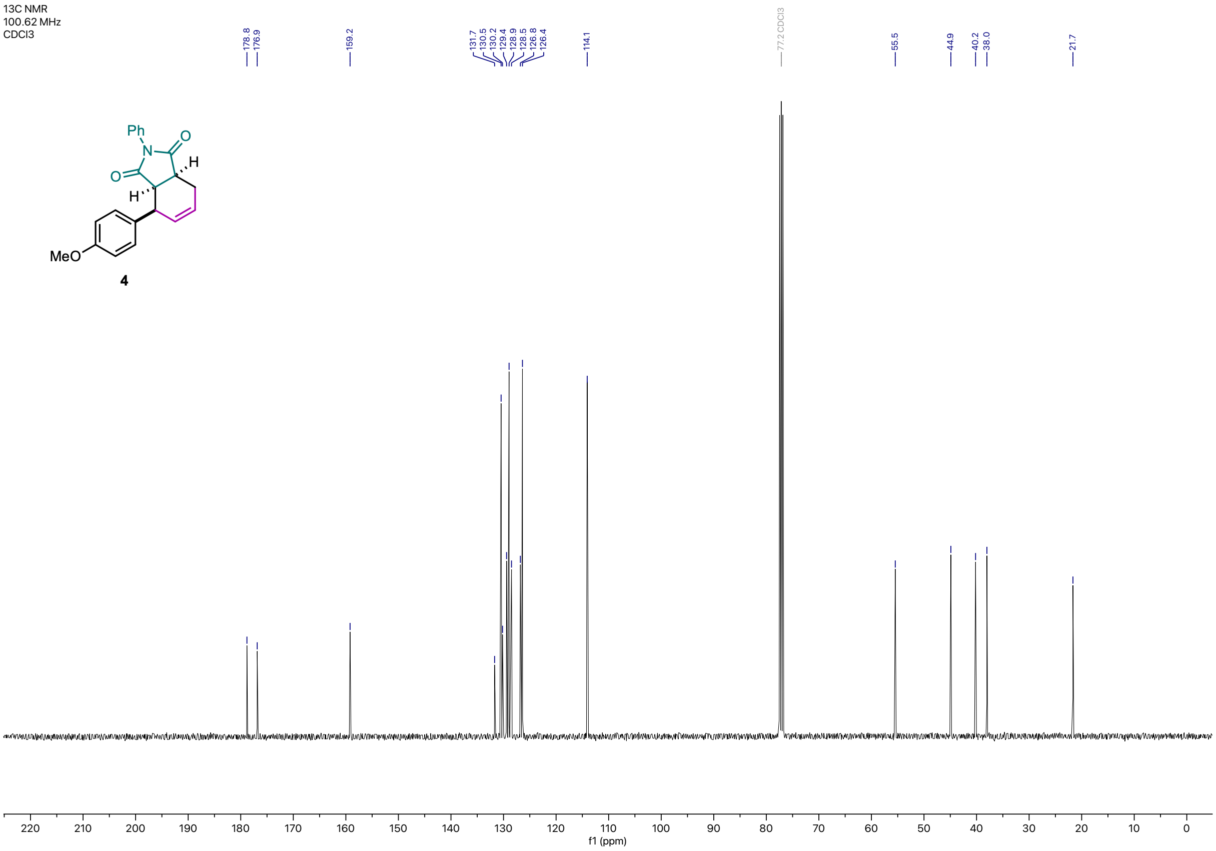

Supplement: MR-OLF-D6MR00016A-s001 [file MR-OLF-D6MR00016A-s001.pdf]
